# When evidence is not enough: A qualitative exploration of healthcare workers’ perspectives on expansion of two-way texting (2wT) for post- circumcision follow-up in South Africa

**DOI:** 10.1371/journal.pdig.0000867

**Published:** 2025-06-05

**Authors:** Isabella Fabens, Calsile Makhele, Nelson Igaba, Khumbulani Moyo, Felex Ndebele, Jacqueline Pienaar, Geoffrey Setswe, Caryl Feldacker

**Affiliations:** 1 International Training and Education Center for Health, Department of Global Health, University of Washington, Seattle, Washington, United States of America; 2 Aurum Institute, Johannesburg, South Africa; 3 Right to Care, Pretoria, South Africa; 4 Department of Health Studies, University of South Africa, Pretoria, South Africa; 5 Department of Global Health, University of Washington, Seattle, Washington, United States of America; The University of Hong Kong, HONG KONG

## Abstract

As per South African national guidelines, in-person follow-up visits after voluntary medical male circumcision (VMMC) are required but may be unnecessary. Two-way texting (2wT), an mHealth platform, engages clients in post-operative care and triages those with complications to in-person review. 2wT was found to be safe, effective, and efficient. In South Africa, to understand provider perspectives on 2wT and potential for expansion, 20 key informant interviews were conducted with management, clinicians, data officials and support staff involved in 2wT scale-up. Interviews were analyzed using rapid qualitative methods and informed by two implementation science frameworks: the Reach, Effectiveness, Adoption, Implementation and Maintenance (RE-AIM) framework and the Pragmatic, Robust, Implementation and Sustainability Model (PRISM). Participants shared mixed and multi-faceted feedback, including that 2wT improves monitoring and evaluation of clients and clinical outcomes while also reducing follow-up visits. Challenges included duplicative routine and 2wT reporting systems and perceptions that 2wT increased workload. To improve the likelihood of successful 2wT scale-up in routine VMMC settings, participants suggested: further 2wT sensitization to ensure clinician and support staff buy-in; a dedicated clinician or nurse to manage telehealth clients; improved dashboards to better visualize 2wT client data; mobilizing 2wT champions at facilities to garner support for 2wT as routine care; and updating VMMC guidelines to support VMMC telehealth. As attendance at follow-up visits may not be as high as reported, implementing 2wT may require more effort but also brings added benefits of client verification and documented follow-up. The transition from research to routine practice is challenging, but use of RE-AIM and PRISM indicate that it is not impossible. As VMMC funding is decreasing, more effort to share the evidence base for 2wT as a safe, cost-effective, high-quality approach for VMMC follow-up is needed to encourage widespread uptake and adoption.

## Author summary

South Africa has a high prevalence of HIV, and voluntary medical male circumcision is a prevention method recommended by the World Health Organization. Two-way texting (2wT) is a digital health intervention that allows males to receive educational messages after circumcision about healing and send messages directly to a provider via SMS or WhatsApp. This system detects as many complications as routine follow-up care, though is more efficient. As we scale up to new locations in South Africa, we wanted to ensure the approach is well-adapted for health workers. Through 20 interviews with health workers, we found that it saves clients in-person visits and helps with data collection. However, more effort is needed to introduce the intervention to clinicians, including providing better training and appointing champions to help motivate clinicians. Funding for HIV and VMMC programs is decreasing, so it is important to find tools such as digital health to reduce the burden on facilities while maintaining quality client care.

## Introduction

In South Africa, 17% of those ages 15–49 had HIV as of 2023 [1]. Voluntary medical male circumcision (VMMC) is one of the many interventions recommended by the World Health Organization to reduce transmission of HIV [2] as it has been found to be 60% effective in reducing transmission from women to men. A nationally representative survey conducted in 2017 found 62% of males to be circumcised in South Africa [3]. Although VMMC is a safe procedure with few adverse events [4–9], global VMMC guidelines still require clients to attend two post-operative follow-up visits within 14 days to ensure timely identification and treatment for adverse events. This presents an undue burden for providers and clients [10,11] creating potential barriers to continued VMMC expansion in support of HIV prevention efforts. Therefore, in 2018, the International Training and Education Center for Health at the University of Washington implemented two-way texting (2wT) to provide SMS-based telehealth in Zimbabwe, ensuring a safe and more efficient option for VMMC follow-up [12–14]. In 2021, the International Training and Education Center for Health partnered with the Aurum Institute, the Centre for HIV-AIDS Prevention Studies, and Right to Care to expand on this work in South Africa, conducting a randomized controlled trial (RCT) [11,15,16]. Similar to Zimbabwe results, 2wT was found effective to provide safe, lower-cost follow-up that greatly benefitted VMMC clients who approved of the approach [11,15–17]. Previous qualitative studies among healthcare workers in both Zimbabwe [13,18] and South Africa [16] found 2wT to be useful, acceptable, and highly usable. However, from the health care worker implementation perspective, challenges were identified in duplicative VMMC reporting systems that increased workload, the need for specific 2wT personnel, and the need for improved client wound care counseling to reduce concerns that clients were unable to identify complications swiftly.

During a subsequent stepped wedge trial on 2wT expansion potential in South Africa [19], our objective was to complement previous findings by focusing on new opportunities for adaptation and identifying additional potential pitfalls moving 2wT from research to routine VMMC settings and service delivery teams. Although rigorous implementation science (IS) studies of mobile health (mHealth) interventions remain rare, we used an IS approach to help understand how, why or for whom this translation of the 2wT mHealth implementation might fail or succeed [20,21]. As part of an expansion study of the impact of 2wT on VMMC follow-up in routine settings in South Africa, we used two complementary IS frameworks to qualitatively evaluate 2wT in routine practice. We applied the RE-AIM (Reach, Effectiveness, Adoption, Implementation and Maintenance) framework alongside its complement, the Practical, Robust Implementation and Sustainability Model (PRISM), to explore factors that facilitate or curtail 2wT expansion potential [22]. The RCT and stepped wedge study outcomes [19] provide strong, quantitative evidence for individual (client) *reach* and *effectiveness* within the RE-AIM framework [22]. Through this qualitative paper, we aimed to explore 1) *implementation* (RE-AIM) at the clinician and organizational levels to identify features for improved client, provider, and organizational experience; 2) *internal context* (PRISM) to understand how to effectively work within the routine context of staffing structures and client preferences; and, 3) *external context* (PRISM) that may influence 2wT maintenance such as guidelines and targets.

## Materials and methods

### Qualitative reporting guidelines

We included applicable components from the Consolidated Criteria for Reporting Qualitative Research (COREQ) [23], a comprehensive reporting framework for qualitative research. To enhance trustworthiness in our results, we report our methods for data collection and analysis using four criteria: credibility (the results presented are an accurate depiction of the phenomenon), transferability (include details that would allow other researchers to compare to other contexts), dependability (include details so the study could be, in theory, replicated) and confirmability (results are from participants, not the researchers) [24]. Specifically, details on routine delivery, expansion trial and 2wT support transferability are covered in the data collection section; the analysis section speaks to dependability; and the positionality section attests to credibility in terms of details on the research team.

### Routine VMMC service delivery

The US President’s Emergency Plan for AIDS Relief (PEPFAR) recommends VMMC for males ages 15 and older across 15 priority countries in Eastern and Southern Africa as a cost-effective component of comprehensive HIV prevention [25]. Post-VMMC follow-up at least once within 14 days is required [26], though PEPFAR specifically endorses 2wT for low-risk clients [25]. All VMMC care, regardless of follow-up method, is provided free in National Department of Health facilities in accordance with VMMC guidelines [27]. Clients are asked to return for scheduled day 2 and day 7 visits; some clinics or teams provide transport for clients unable to return for review. Clients typically return to the same facility where they were circumcised for follow-up; however, in emergencies or by choice, clients may seek free care at any National Department of Health facility. VMMC teams at circumcising facilities consolidate data across sites during monthly data verification activities. Sites used the standardized PEPFAR approach to assess, identify, and record the timing, type and severity of adverse events [28]. For clients who miss their first post-operative visit two days after circumcision, follow-up tracing is conducted via phone call. VMMC teams are expected to report key PEPFAR outcomes: VMMC productivity by age group; adverse events by severity and type; and number of VMMC clients with at least 1 follow-up visit within 14 days [26]. In addition to National Department of Health routine reporting forms, some implementing partners, including Right to Care, collect additional client M&E data from their clinician and clerical teams.

### 2wT expansion trial

We conducted a modified stepped wedge study from January 30 – October 19, 2023, in Ekurhuleni District (Gauteng Province) and Dr. Kenneth Kaunda District (North West Province), South Africa [29]. As 2wT rolled out, clients during intervention periods were able to opt in to 2wT via Short Message Service (SMS) or choose standard of care (in-person visits). Clinicians retained discretion on 2wT enrollment; it was not always offered at all sites. Between August 17 – October 19, only, WhatsApp was offered in addition to SMS as a platform for 2wT communication.

### 2wT technology and implementation

2wT has been described in detail previously [11,12,19,30]. In brief, 2wT is a hybrid mHealth system that combines automated and personalized messages between clients and a VMMC nurse on SMS or WhatsApp. Clients could choose between Afrikaans, English or Zulu on either platform. Those using SMS also had the options of Sotho and Tswana. Males ages 15 and above with a phone present at their VMMC appointment were eligible to enroll for the opt-in 2wT follow-up approach. On the day of VMMC, clients were educated about using 2wT in lieu of scheduled post-operative visits and given a pamphlet with instructions and emergency contact numbers. 2wT clients were told they were welcome to visit the clinic at any time, in addition to using 2wT. After discharge, on Days 1–3, 5, 7, 10 and 13, 2wT participants were sent automated messages in the language they chose asking them to respond about complications like bleeding, swelling, or wound opening their healing. The 2wT system only recognized text messages; any other content such as emojis or photos were not visible to the system. On the other days (4, 6, 8, 9, 11 and 12), they were sent educational messages. If no response was received by day 3 for minors or day 8 for adults, a nurse followed up to trace the clients and ensure healthy healing.

2wT was implemented as a hub (central nurse) and spoke (facilities) model for the stepped wedge study [31]. Facilities were routine VMMC service delivery sites where non-study teams performed VMMC procedures and counseling according to National Department of Health standards. Facilities recruited, enrolled, and educated clients on 2wT and reviewed 2wT clients who needed follow-up care. The central nurse was a study-specific VMMC nurse who responded to clients, called clients who did not respond, and referred clients to facilities for follow-up care. When clients responded to the system with a concern, the central nurse responded, triaged, and referred to facilities if needed. Clinicians from facilities were asked to check 2wT and respond to messages in addition to other circumcision duties. Facility staff were responsible for filling out paper forms for routine and 2wT clients. The central and facility nurses accessed the 2wT system and messaged clients on work tablets or laptops for ease of use in monitoring various clients. Facility and central nurses were instructed to use their work phones for all activities external to 2wT system communication, including tracing or phone-based interaction at the clients’ request.

### Conceptual model

**Fig 1:**
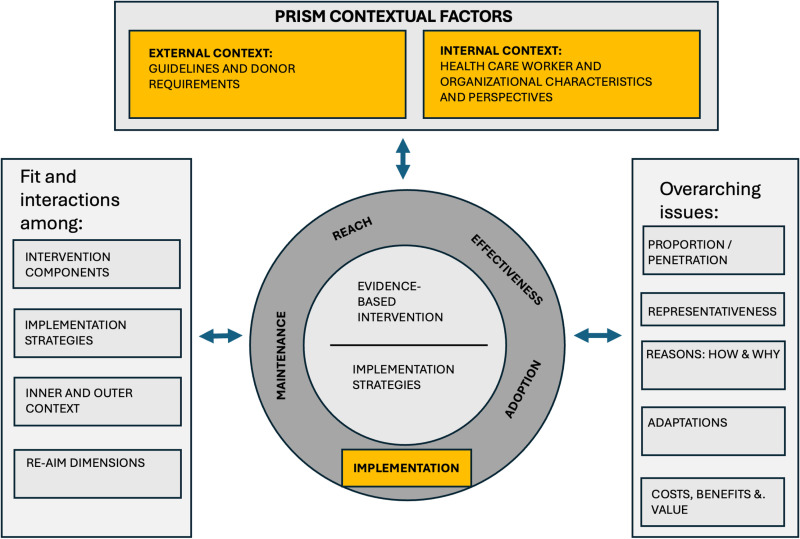
Adaptation of the Practical, Robust Implementation and Sustainability Model (PRISM) and Reach, Effectiveness, Adoption, Implementation and Maintenance (RE-AIM) Frameworks* for the stepped wedge study. *Model adapted from: Glasgow et al. (2019) [32] that expanded upon the PRISM framework (Feldstein & Glasgow, 2008) [35]. Constructs in yellow were explored in this study.

The conceptual model and qualitative approach were created using two common IS frameworks to guide successful translation of research to routine practice. RE-AIM [22,32] was operationalized at the staff/setting levels (implementation and adoption) to guide measurement of what, where, when, and how 2wT was implemented (Fig 1) [33,34]. KIIs with 2wT managers, clinicians, the M&E data team and support staff identified insights into how to improve 2wT and its implementation. The *Adoption* portion of the RE-AIM framework includes the number and proportion of staff at facilities that implemented the intervention. We did not quantitatively measure how many staff adopted 2wT, though our findings reflect reasons why someone may or may not have adopted 2wT. Second, the study team incorporated aspects of PRISM [35] to inform actionable recommendations, including internal fit (health care worker and clinical context) and external context (South African VMMC policies and guidelines) [36]. The combined RE-AIM and PRISM-informed model [32] (Fig 1) shaped the interview guide, analysis, and recommendations.

### Data collection and analysis

Key informant interviews (KIIs) were conducted from November 20, 2023, to January 19, 2024. Using the conceptual model, the study team developed a semi-structured interview guide which was pilot tested through an initial practice interview in which the participant critiqued the guide. The study team made subsequent improvements. To recruit interview participants, the study team informed facility staff involved with 2wT implementation during an in-person support visit or via email. Facility staff included clinicians, managers, the M&E team and support staff members. The central nurse was not part of the routine VMMC implementation team and was excluded. All invited staff volunteered to be interviewed and consented before being interviewed in a private room of the facility or at the study team office if more convenient for the respondent. One interview with a health facility staff member not involved in 2wT was conducted; this interview was excluded as they were not a direct user of the system. Three multi-lingual interviewers (including authors CM and FN) conducted interviews in English; participants also responded in IsiZulu, Sesotho or Setswana. Only the interviewer (in some cases, two of the interviewers) and the participant were present for each interview. Interviews were recorded and took between 12 minutes and 1 hour 23 minutes, an average of 53 minutes.

Interviews were transcribed and translated into English. Authors CM (employed by Aurum) and IF (employed by University of Washington) analyzed interviews using rapid qualitative analysis [37,38]. The analysts wrote 2-page summaries of each interview, created a list of themes from summaries and prepared the results using a matrix in Google sheets to inform recommendations.

### Positionality and reflexivity statement

The study team consisted of two researchers at University of Washington, four at Aurum and two at Right to Care. Three researchers from Aurum conducted the interviews, two of whom are authors. The lead interviewer was author CM, a black African female who has a master’s degree and was directly involved in implementation. CM was known to many of the participants as the Study Coordinator, so there was increased rapport which could influence social desirability bias [39,40] in both positive and negative ways [41,42]. The other two interviewers were not involved in the day-to-day 2wT implementation, nor did they know the interviewees. The confidentiality statement read to individuals stated the reason for the qualitative study and emphasized confidentiality of data during and after collection, which are strategies to encourage accuracy of responses [41]. None of the authors were primarily based at a facility implementing 2wT, which could mean the study team could more objectively collect and analyze data or that they were missing context specific to working at the facility. While attention was given to maintain objectivity, the research team was involved in conducting and analyzing KIIs, a bias that cannot be quantified.

### Ethics

The review boards of University of Washington (No. 00009703; CF) and the University of Witwatersrand, Human Research Ethics Committee (No. 200204; GS) approved the study protocol for the routine scale-up of 2wT as part of the stepped wedge study. KII participants completed a written informed consent. No personal identifiers were included in the analyzed data.

## Results

**Fig 2:**
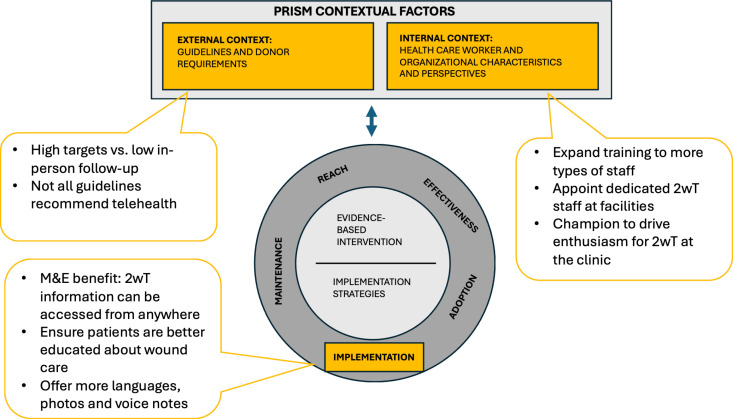
Results within the context of RE-AIM and PRISM*. *Model adapted from: Glasgow et al. (2019) [32] that expanded upon the PRISM framework (Feldstein & Glasgow, 2008) [35]. Constructs in yellow were explored in this study.

Interviews from 20 participants were included in analysis (Table 1). Using the conceptual model, findings are presented in three construct groupings: 1) implementation, including factors related to adoption; 2) 2wT internal program fit factors including training and staffing of clinicians and support staff; and 3) the external fit to South African context, including VMMC policies and guidelines (Fig 2).

**Table 1:** Participant job categories.

Type of Employee	Number
Management	4
Clinicians	6
M&E team	8
Support staff	2
**Total**	**20**

### Implementation

#### 2wT as a tool for monitoring and evaluation (M&E).

Many participants (4 clinicians, 3 M&E staff and 2 managers) noted advantages of 2wT for M&E. Some participants understood that 2wT improves follow-up verification, reporting and planning, including that it provides a record of client care, improves time management and increases communication among clinicians. Other participants noted that 2wT allows clinicians to address tasks at their own pace and document client care within the system. They added that the system also captures client details, so information can be accessed by teams from any location. Before 2wT, a participant reported that when males could not afford transportation to the VMMC clinic, they could be reviewed at their nearest clinic with data on local paper forms, resulting in gaps in client records. Participants also appreciated improved communication between clients and providers, remarking that clients could ask their providers questions at any time via SMS, filling in potential gaps in education. One clinician used the 2wT system to message with clients and arrange appointments during routine business hours. In addition, participants thought that 2wT could improve adverse event reporting, since males in routine care seldom returned for their follow-up visits.

*“Some of them would sit and not even want to come in. If they hadn’t texted via [2wT] we would not even know, because sometimes you would call a client and they’ll say no, I’m fine. And the client is not okay. So, when it’s a red flag on that side, then we can eventually communicate with them. So yeah, it did help a lot.” [Participant 16, M&E Team Member]*

However, four participants (2 M&E staff and 2 managers) also proposed ways to improve 2wT for routine M&E. First, a manager admitted that they do not look at 2wT reports, but it would be helpful for them to know how many 2wT clients are not coming back for follow-ups, a query that a clerk could answer using the 2wT system. Another M&E staff member reported that clinicians were not following up with clients, noting: *“I wish the raw data thereof could be linked to our system so that we have an updated dashboard. [Participant 21, Manager]* This suggested dashboard could inform monthly meetings with the M&E manager and senior management to improve decision making. A linkage between 2wT and the current data visualization tools would also be beneficial.

#### Reduce electronic and paper parallel reporting systems.

Despite noted benefits of 2wT for M&E, nearly half of participants (2 clinicians, 6 M&E staff and 1 manager) noted an added burden of parallel reporting systems as clinicians are required to report all clients, including 2wT clients, on routine National Department of Health forms. This documentation duplication between the digital and paper systems greatly reduced enthusiasm and buy-in. Another challenge was that when paper forms were not filled out, it delayed reporting to the funder. Furthermore, there were ways in which 2wT and paper forms did not align; if a client messaged on 2wT that they had a potential healing issue, that needed to be recorded on the paper forms, though the clinicians had a hard time classifying an adverse event without seeing the wound. One participant noted a possible solution is that 2wT could replace some of the required National Department of Health paper documentation.

*“I think the amount of writing whether it’s [2wT] or follow up on paper, eventually becomes heavy on anyone. But if I was just focused on the 2-way texting, already [the provider] and the clients have typed something.” [Participant 21, Manager]*

#### Apply 2wT eligibility criteria consistently for appropriate client enrollment.

Over time, providers noted that they learned to be more selective in who they enrolled into 2wT and who they required to attend in-person reviews. A few providers (2 clinicians and 1 M&E staff member) mentioned experiences with minors that led them to stop enrolling them or do so under more scrutiny. For example, some minors do not have a phone and use their parent or guardian’s phone, so the guardian needs to be home with the client and attentive to the messages. Also, clinicians were concerned about allowing clients to enroll via WhatsApp because clients might not have a data plan, instead requiring clients to enroll using SMS which was free. Other types of clients for whom the providers exercised caution were those with existing non-VMMC related medical complications or clients without access to water to wash their wound, presenting hygiene concerns. Overall, the need to consider each client, individually, and match 2wT to the client was considered prudent.

*“It is very convenient, but for other age groups especially minors, we want to monitor them because sometimes the doctors struggle to cut them. Sometimes I feel that if the client does not come back in person, we may miss an adverse event. Their mother will just pour water but doesn’t bathe [their child]. [Clients may] get [an] infection or the mother will use home remedies.” [Participant 1, Clinician]*

#### Ensure 2wT clients are well educated in adverse event identification.

Many participants (7 clinicians, 4 M&E staff, 1 support staff and 1 manager) recommended improvements for client education. First, support staff members who recruit clients should tell males to bring their phone or know the phone number of the person whose phone they intend to use. Some requested more client education materials such as leaflets to hand out or a video to educate clients. Clients should also be aware of what to expect regarding wound care, for instance, when to remove their bandage and to expect a small amount of bleeding. Finally, there are interpersonal considerations. For instance, males may not feel comfortable receiving wound care information from a woman or may be afraid to come back to the clinic if they have an AE. Specifically, for 2wT, males should be coached on how to respond in a way that the system can understand responses; some respond with the letter O instead of the number 0. Males should also be reminded to respond even when they do not have an issue, so the clinical team knows they are fine.

Although the efficiency and safety benefits of 2wT were noted most participants (3 clinicians, 4 M&E staff and 1 manager) who felt 2wT increased their ability to identify adverse events, others expressed concerns that using this telehealth approach (1 clinician, 1 support staff member, 2 M&E staff and 1 manager) could miss adverse events. One participant recommended that clients first come for their check-in two days after circumcision and then start 2wT because they felt they were abandoning their clients. Other clinicians feared that post-operative client education was not strong enough so clients would not be able to correctly identify signs of complications.

*“Because the [client] is not a qualified somebody, [they will] not be able to pick it up because the [client] will say there is no adverse event. And the [central] nurse would say, ‘no there is no adverse event’. But you find that there is an infection hiding somewhere. Only a qualified [clinician] can actually see that something is not 100 percent.” [Participant 10, M&E Team Member]*

#### Enhance 2wT functionalities.

Most participants (7 clinicians, 6 M&E staff, 1 support staff member and 1 manager) recommended additional functionalities for 2wT, including offering the routine texts in more languages to better include clients from neighboring countries who speak different languages. Participants also recommended adapting the system to allow clients to send photos or submit voice notes, reducing client literacy or language barriers. Finally, a suggestion for improved notifications was to include sound alerts when new messages came in from clients, especially for those submitting potential adverse events.

*“… especially when it comes to the foreigners trying to [text in] Zulu, they will select Zulu because they are not good [at] English like [especially] from Mozambique, they [speak] Portuguese but they can speak Zulu and some are even very fluent. But when it comes to writing it down … we just struggle to understand … [what this person is saying]. [I] preferred even calling most the time. [Participant 1, Clinician]*

Besides offering more languages, photo capability and voice notes, there were some small changes requested for the system itself. Specifically, participants suggested adding country code validation for phones numbers; a field for emergency contact; a designation to separate smartphones and basic phones; and the ability to filter messages by client. They also suggested recording common voice messages to clients to reduce the number of phone calls providers had to make and giving the clients an option to choose what time they received automated messages, which would be more convenient for clients who work.

### Internal fit factors

#### Offer hands-on training and mentoring to health workers to ensure confidence in 2wT use.

Most participants (5 clinicians, 4 M&E staff and 1 support staff) found the 2wT trainings to be helpful, especially the practical elements in which trainers demonstrated how to use the system. One participant in a support staff role (recruits and finds non-responsive clients for follow-up) noted they wanted to be more involved in trainings, *“I would like to have a proper training now … even though I’m [in] the field … I must be empowered” [Participant 2, Support Staff].* Half the KII participants requested training for more types of staff, including support staff and nurses replacing those on leave. For instance, a support staff member wanted to know what to do if a client was bleeding, or more details on WhatsApp as an option for follow up. Furthermore, staff who were not trained gave misinformation such as the fact that all 2wT clients had to come back for in-person follow-up visits. Participants preferred 2wT-specific mentoring, including in-person check-ins or virtual support through phone or video calling.

*“We need to do some in-service training amongst ourselves, just to remind ourselves which aspects are more [important], what basis we need to touch on during the in-service training. We sometimes have weekly meetings where we sit down and talk and look at the aspects. I think those meetings can be [improved].” [Participant 8, Clinician]*

#### Appoint dedicated staff for 2wT as part of routine VMMC care.

Several participants (2 clinicians, 3 M&E staff and 1 manager) mentioned that 2wT was easy to use, but two participants (1 clinician and 1 M&E staff member) noted comfort with the system increased over time. One expressed their slower uptake: *“We are also still new on this 2 way texting. We are being resistant to change, but I think it will work better with time [Participant 19, M&E Team Member].*

While participants adapted to using the system, they found it burdensome to manage on top of clinical reporting duties. To speed expanded 2wT use at site level, many participants (5 clinicians, 6 M&E staff, 4 managers and 2 support staff) recommended 2wT should have dedicated staff at facilities to reduce the perceived 2wT workload as additional to routine VMMC duties.

*“If it’s just us and there are too many [clients], we suffocate and there’s nothing we can do. One day we had 21 [clients]. [There were only two of us] and then imagine [us] having to cut and enroll. And then assuming that each [client] is five minutes, that’s more than an hour. Then were like, okay, let’s cut everyone then we will enroll after. … And we ended up unable to enroll the [clients] unfortunately, because after 6, [clients] had to go home.” [Participant 8, Clinician]*

#### Identify a 2wT champion to encourage adoption of 2wT.

While many participants requested dedicated 2wT staff, others felt that a 2wT clinician or staff champion was missing to drive internal clinic enthusiasm. Some considered 2wT to be an external study, not a component of routine VMMC care, with one person stating that 2wT is *“still a study, so we don’t even know if this thing works or not. But you guys are already forcing it on us.” [Participant 14, Manager]*. Another participant identified that when only one person at the clinic uses 2wT, its implementation stops when that person leaves. That same respondent noted that a champion was needed to promote the advantages of 2wT and encourage adoption at the clinic.

*“What we could not master is how we should be benefiting actually from 2wT. Especially because of not having the person [who] champions [2wT]. I think at some facilities you were able to see that at least this person is the one that is doing it. But as soon as that person is not there, then there’s no one who takes over.” [Participant 18]..” [Participant 18, Manager].*

### External context

#### Leverage client preferences for expansion support.

Participants mentioned several benefits for clients in addition to those noted for staff. Many of the participants mentioned males do not want to come back to the clinic for follow-ups because they think it is unnecessary, do not have enough money to pay for transportation or are afraid of being seen at the clinic by others in the community. Part of this is related to the fact that circumcision is traditionally a private matter, so they do not want people to see them at the clinic and assume they are getting circumcised, “*Sometimes we as nurses we feel … [uncomfortable] …That you have to go to the clinic [because] people can see you*.” *[Participant 2, Support Staff].* 2wT offers a private way to communicate with providers without the issue of being seen at the clinic,

“*If a client is alone, they are more comfortable when they’re in their own space so they can actually text. … a client would rather be there and be ill [rather than] coming in.” [Participant 16, M&E Team Member]*

Aside from the fact that 2wT can reduce in-person visits, participants mentioned types of clients for whom the intervention was more useful, specifically, those who live in urban areas or younger males because they already have phones. 2wT may also offer benefits to youth and men who work or travel. A participant noted that 2wT is popular among males because system is easy to understand. *“A 10-year-old would be able to use it [2wT], because it asks you a question you either answer with zero or you answer with a one.” [Participant 16, M&E Team Member]*. Finally, one person said 2wT could be promoted via social media and the radio, and clients could be given items to take home to encourage them to get circumcised.

Moreover, some participants noted that the addition of WhatsApp alongside SMS helps reach clients with smartphones who use WhatsApp more frequently while not excluding those in informal settlements or of lower socio-economic status who may only have basic feature phones. Some who recommended WhatsApp did so because it has the capability to send photos, and it is faster than SMS.

#### Align external guidelines and targets with evidence.

A few participants (3 M&E staff, 2 clinicians and 1 support staff member) noted that completing all required in-person reviews was challenging. In part, many clients faced transportation issues or clients attending their day 2 follow-up visit would not complete additional follow-up visits. These realities, combined with the pressure to report adherence to required visits for the funder, could lead to overreporting of post-operative visit attendance. One respondent noted they felt obligated to report near 100% follow-up even if many clients were unable or chose not to return for in-person visits.

“*We would always have problems when we report to the funder, because the documents will not be filled. But the system will be saying 100% or 98%. If it’s not documented, it means it’s not done*.” *[Participant 10, M&E Team Member]*

One participant mentioned that enrolling clients in 2wT conflicts with the National Department of Health guidelines that require in-person visits, resulting in low support for 2wT uptake and expansion. They were concerned about promoting an approach that contrasted to the in-person review requirement.

*“In [District X], I know the Department of Health does not like 2wT. I don’t know if it’s because, according to the guidelines, [we] need to physically see the [clients]. [Participant 14, Manager]*

## Discussion

Conversations with participants including clinicians, M&E data officers, VMMC managers and support staff demonstrate the complexity of moving from research to routine practice for evidence-based mHealth innovations. Overall, interviewees reported several facilitators of expansion, including that 2wT increases timely communication between clients and providers; fills potential gaps in client education; improves follow-up verification; and strengthens reporting. However, implementation was stymied by several obstacles at both provider and organizational levels, including perceptions of increased workload, lack of site champions and lack of an enabling policy environment. With competing demands on clinicians’ time and decreasing global VMMC funding, evidence-based, effective, safe, and cost-effective mHealth innovations like 2wT should be attractive at client, provider, and organizational levels. However, 2wT obstacles appear more influential than perceived benefits, reducing buy-in and momentum to scale. Application of the RE-AIM and PRISM frameworks shed light on several opportunities to energize 2wT buy-in and facilitate scaling across South Africa. Table 2 summarizes recommendations from the study team with examples of sentences in the results section to justify them.

**Table 2:** Recommendations and their justifications by theme.

Recommendation	Theme	Justification from results section
Training to emphasize that 2wT augments, not replaces routine care	Implementation/ Apply 2wT eligibility criteria consistently for appropriate client enrollment	“Overall, the need to consider each client, individually, and match 2wT to the client was considered prudent. ‘*It is very convenient, but for other age groups especially minors, we want to monitor them because sometimes the doctors struggle to cut them.’ [Participant 1, Clinician]”*
Training to focus on the idea that 2wT decreases in-person care but may not reduce overall workload	Internal fit factors/ Offer hands-on training and mentoring to ensure confidence in 2wT use	To speed expanded 2wT use at site-level, many participants … recommended 2wT should have dedicated staff at facilities to reduce the perceived 2wT workload as additional to routine VMMC duties.
Training curriculum should include the published evidence that the 2wT system is safe and efficient	Internal fit factors/ Identify a 2wT champion to encourage adoption of 2wT	“*We don’t even know if this thing works or not. But you guys are already forcing it on us” [Participant 14, Manager]*
Staffing and accountability structures to be improved through additional 2wT staff at sites, or routine staff “champions”	Internal fit factors/ Identify a 2wT champion to encourage adoption of 2wT	*I think at some facilities you were able to see that, at least this person is the one that is doing it. But as soon as that person is not there, then there’s no one who takes over.” [Participant 18, Manager].*
In-person, post-operative visit targets to be adjusted for 2wT telehealth approach	External context/ Align external guidelines and targets with evidence	One respondent noted they felt obligated to report near 100% follow-up even if many clients were unable or chose not to return for in-person visits.
Guidelines to include support for evidence-based telehealth interventions like 2wT	External context/ Align external guidelines and targets with evidence	*“In [District X], I know the Department of Health does not like 2wT. I don’t know if it’s because, according to the guidelines, [we] need to physically see the [clients]. [Participant 14, Manager]*

Our study team noted several items that should be improved for future implementations of mHealth interventions, including areas to emphasize for 2wT clinician training. First, trainings should include that the RCT in South Africa proved 2wT to be safe and efficient [11,15,17]. Without knowledge of the RCT outcomes, providers lacked confidence that 2wT could ensure quality care with referral and tracing safeguards. Second, 2wT augments, not replaces, in-person visits. Site teams needed more confidence to exercise their discretion to recommend low risk clients to 2wT and schedule minors or clients with higher perceived adverse event risk to in-person reviews. Moreover, there was frequent staff turnover, so staffing plans should ensure that new and replacement staff are also trained on 2wT. Lastly, efforts should increase focus on 2wT’s benefit of providing quality follow-up with fewer in-person reviews, rather than promote the reduction in overall VMMC workload [43]. Alongside efforts to promote 2wT benefits for clinicians and client care, more emphasis is needed to increase awareness of 2wT benefits for data quality and client M&E. Additional, targeted training for the M&E staff and managers on real-time 2wT dashboards with complete client data could further build support for 2wT’s effect on data quality, garnering more manager support for the reporting benefits. At the organization level, lack of awareness of 2wT benefits for accountability, decision-making, and client verification was also evident.

While training is important to emphasize the benefits of 2wT and its use for streamlined management, staffing and accountability structures should be adapted to promote buy-in and sustainability. In this study, the central nurse was a strong promoter of the system and drove the creation of tasks for facilities to resolve. In the future, facility teams, themselves, would need increased encouragement and supervision to complete tasks on time, trace clients and update 2wT. To create more enthusiasm for 2wT clinical and M&E advantages, and support expansion planning, a champion from a high 2wT uptake site should be engaged in a peer-to-peer support for 2wT adoption. These champions, either central or facility-based, could reinvigorate 2wT enthusiasm and consideration of 2wT as a locally optimized intervention [44,45], increasing the likelihood of successful scale-up. Additionally, integrating 2wT performance metrics into facility-level quality improvement processes could help ensure continuous monitoring and optimization, making 2wT adoption more sustainable. Finally, institutional support from leadership—including policy alignment, workload redistribution, and incentives for early adopters—could further strengthen engagement and ownership. This would increase the likelihood of successful scale-up and ensure that 2wT becomes a standardized and self-sustaining intervention in routine VMMC care. Through our dissemination, we have introduced 2wT to implementing partners, and some are keen to implement it even for other health areas; this paper presents lessons learned that can be used in future uses of 2wT.

In addition, participants noted confusion caused by inconsistencies between external policies and 2wT as well as follow-up requirements. For instance, fewer in-person follow-up visits likely happen than reported, creating a potential false workload comparison between 2wT and routine care visits that reduces the pressure for policy change. Reported VMMC follow-up rates vary, with studies finding between 63–90% of VMMC clients attending at least one visit [11,46–48]. However, over-reporting of follow-up visits may reflect fear of consequences, including job loss or other disciplinary measures [49]. Funders must be aware of the potential for over-reporting when setting targets and should work with implementing partners to develop targets that match the true number of follow-up visits that currently occur. For better understanding of how many required follow-up visits are missed, those who guide project planning should invest in data quality assessments [50].

Our findings fill several gaps in the literature including that providers are concerned with mHealth workload [51] and that provider acceptability for mHealth must go beyond RCTs to evaluation in routine settings [51,52]. Also, these findings illuminate some contextual factors surrounding adoption, a gap identified in the 2019 review of the RE-AIM Framework [32]. Finally, one of the motivators for the creation of the PRISM framework was the over-emphasis on success among program recipients without examining challenges for staff [35]; this paper aims to broaden understanding of the successes and challenges of 2wT from the point of the healthcare workers.

### Strengths and limitations

First, the addition of this paper complements the previous manuscripts reporting on the stepped wedge trial, including 2wT reach and effectiveness [19], the 2wT messaging platform, itself [31], and cost drivers [53], presenting a broad and balanced evaluation approach of this digital innovation in routine settings in line with PRISM guidance [35]. The 2wT study team also interviewed all staff directly involved in implementation; thus, we ensured as many perspectives were captured as possible, including from clinicians and non-clinicians. However, although we included all direct implementers of 2wT, members of the National Department of Health were not included in KIIs. Future efforts would benefit from inclusion of their perspectives. Client perspectives [29] and common constraints of electricity, network, and funding [16,18] were previously explored and not repeated here. Some clinicians in South Africa and in other low-resource settings use their personal devices for client communication, including review of photographs as part of telehealth support. The 2wT system purposefully does not accept images. Some clinicians or clients may have reviewed images as part of a consultation outside the study framework, a practice that could be explored in a subsequent study. Although this study aimed to enhance implementation of 2wT, suggestions to improve routine VMMC service delivery were passed to VMMC teams. The specific suggestions for 2wT features and functionality were shared with the technical team who could enhance 2wT at scale.

## Conclusions

Increased education, sensitization, and awareness is needed to more effectively move 2wT from research to routine practice and create more enthusiasm for expansion. More attention on the clinical and M&E benefits could help create champions for 2wT scale-up, overcoming hurdles in consistent uptake. Adoption of several recommendations could push the balance in favor of successful scale-up. First, augmented education to ensure that clients are prepared to self-monitor must be consistently implemented, ensuring that clients are informed and empowered to heal safely at home. Second, 2wT M&E processes must be streamlined, including fewer redundancies with the routine VMMC reporting alongside a more user-friendly dashboard. Third, there needs to be policy coverage to allow for telehealth (including 2wT) for clients who opt-in, allowing clinician discretion to recommend in-person reviews only when needed. With clear 2wT benefits for clients, clinicians, the M&E data team and VMMC managers, more engagement of these key stakeholders could overcome identified hurdles to 2wT expansion, creating momentum to scale.

## Supporting information

S1 File Construct codebook. This matrix provides details on where text fits within framework constructs, including inclusion criteria, exclusion criteria and example text. This serves a similar purpose to a codebook, though since we applied Rapid Qualitative Analysis, we refer to constructs rather than codes.
(XLSX)

S2 File Informed consent. The informed consent document for interview participants.
(DOCX)

S3 File Interview guide. Semi-structured interview guide for qualitative interviews.
(DOCX)
