# Supplementary material for: When evidence is not enough: A qualitative exploration of healthcare workers’ perspectives on expansion of two-way texting (2wT) for post- circumcision follow-up in South Africa
Source: PLOS Digit Health. 2025 Jun 5;4(6):e0000867. doi: 10.1371/journal.pdig.0000867 (PMC12140269; doi:10.1371/journal.pdig.0000867)
Supplement: S2 File — The informed consent document for interview participants. (DOCX) [file pdig.0000867.s002.docx]

HREC REFERENCE NO: 200204

PROTOCOL NO: AUR2-8-270 2WT

2WT STUDY

Principal Investigators:

Caryl Feldacker, PhD, MPH, University of Washington, Seattle, WA, USA

Geoffrey Setswe DrPH, MPH, The Aurum Institute, Johannesburg

**Funding Source and/or Sponsor:**

National Institutes of Health, 9000 Rockville Pike, Bethesda, Maryland 20892, USA. PI Name: Caryl Feldacker & Geoffrey Setswe: Application ID: 1 R01 NR019229-01A1. 1-866-504-9552 (tty: 301-451-5939) or [commons@od.nih.gov](mailto:commons@od.nih.gov).)

**Study Contact telephone numbers:**

| Name | Position | Phone number |
| --- | --- | --- |
| **24 Hour Urgent Assistance** |  |  |
| Felex Ndebele | Project Manager | 078 149 0446 |

Good day, my name is _______________________ (INSERT NAME), I am a _____________________ (INSERT DESIGNATION) at The Aurum Institute. I would like to invite you to consider participating in an evaluation, entitled “Expanding and Scaling Two-way Texting to Reduce Unnecessary Follow-up and Improve Adverse Event Identification Among Voluntary Medical Male Circumcision Clients in Republic of South” Africa.

**What you should know about this evaluation:**

- We give you this consent so that you may read about the purpose, risks, and benefits of this evaluation.
- The main goal of this evaluation is to help VMMC clients in Ekurhuleni, Dr Kenneth Kaunda, and Dr Ruth Semotsi Mompati districts.
- We cannot promise that this evaluation will benefit you.
- We want to document your opinions about and satisfaction with, the 2-way texting (2WT texting) follow-up method
- You have the right to refuse to take part or agree to take part now and change your mind later.
- Please review this consent form carefully. Ask any questions before you make a decision.
- Your participation is voluntary.

# PURPOSE

You are being asked to be part of an evaluation to assess the implementation of text-based follow-up after circumcision. What we learn from this evaluation will help the National Department of Health (NDOH) decide if and how to scale two-way text-based follow-up for VMMC.

Two-way texting will be implemented for approximately 800 men in all participating districts. Then, we will include up to 20 clinicians at evaluation sites, like yourself, who will also be asked questions about this evaluation and their thoughts on text-based follow-up. As a clinician, you will be asked questions such as what you know and think about the texting method.

You were asked to consider being part of this evaluation because the information you provide in this evaluation, including how satisfied you are with the texting intervention, your experience with taking care of these men via SMS, and any suggestions you have to improve the texting may improve the intervention in the future.

# PROCEDURES AND DURATION

If you decide to participate the following procedures will happen: We will seek to meet you in a private place and seek informed consent from you. If you consent, you will go to a private space within the clinic, and we will solicit your opinions and experiences regarding deploying 2WT. We will ask you questions like, “What were the challenges of the texting system?” The interview will take approximately 30 minutes. With your consent, the interview will be recorded.

**RISKS AND DISCOMFORTS**

Taking part in the evaluation may cause some psychological discomfort because you will be asked to provide your opinions regarding text-based follow-up after surgical circumcision.

**BENEFITS and** **COMPENSATION**

We cannot promise that you will receive any benefits from this evaluation. The study will help the National Department of Health (NDOH) to make male circumcision SMS follow-up stronger and, potentially, more widely available. No compensation will be offered.

# CONFIDENTIALITY

The information you give us will be kept private (in secret). All information we collected during interviews will be locked away or kept on protected computers. No one outside of the evaluation partner will know the results of the SMS input session or your interview. Any information that could be used to identify you will be shared only with your permission and will not be used in any reports from this evaluation. The recordings will be transcribed without identifiers. The voice recordings of the interviews will be destroyed one year after the activity ends. However, the link between your identifier and the transcripts will be destroyed after the records retention period required by the Aurum Institute and the University of Washington in accordance with the law. These records will be kept locked in a separate file cabinet that only evaluation staff can enter.

The Aurum Institute, Wits HREC, US Government, or University of Washington staff sometimes review studies such as this one to make sure they are being done safely and legally. If a review of this evaluation takes place, your records may be examined. The reviewers will protect your privacy. The evaluation records will not be used to put you at legal risk of harm. A description of this clinical trial will be available on http://www.clinicaltrials.gov, as required by U.S. Law. This Web site will not include information that can identify you. At most, the Web site will include a summary of the results. You can search this Web site at any time.

We have a Certificate of Confidentiality from the United States from the National Institutes of Health. These protections only apply to data held in the United States. This helps us protect your privacy. The certificate means that we do not have to give out information, documents, or samples that could identify you even if we are asked to by a court of law in the United States. We will use the Certificate to resist any demands for identifying information.

We can’t use the Certificate to withhold your research information if you give your written consent to give it to an insurer, employer, or other person. Also, you or a member of your family can share information about yourself or your part in this research if you wish.

There are some limits to this protection. We will voluntarily provide the information to:

- a member of the United States government who needs it in order to audit or evaluate the research.
- individuals at the institution(s) conducting the research, the funding agency, and other groups involved in the research, if they need the information to make sure the research is being done correctly.
- individuals who want to conduct secondary research if allowed by federal regulations and according to your consent for future research use as described in this form.
- to relevant authorities as required by other Federal, State, or local laws.

The Certificate expires when the NIH funding for this evaluation ends. Currently, this is 31 March 2025. Any data collected after expiration is not protected as described above. Data collected prior to expiration will continue to be protected

**ETHICAL APPROVAL:**

• This clinical evaluation protocol has been submitted to the University of the Witwatersrand, Human Research Ethics Committee (HREC) and written approval has been granted by that committee.

• The evaluation has been structured in accordance with the Declaration of Helsinki (last updated: October 2013), which deals with the recommendations guiding doctors in biomedical research involving human participants. A copy may be obtained from me should you wish to review it.

**FUTURE USE**

The information that we obtain from you for this evaluation might be used for future studies. We may remove anything that might identify you from the information and specimens. If we do so, that information may then be used for future research studies or given to another investigator without getting additional permission from you. It is also possible that in the future we may want to use or share evaluation information that might identify you. If we do, a review board will decide whether or not we need to get additional permission from you.

# VOLUNTARY PARTICIPATION

It is up to you whether you want to be part of this evaluation. Your alternative is to not participate in the evaluation. If you decide to be in it, you may stop at any time. These decisions will not affect your employment or future relationship with the NDOH or its partners. If you decide to leave the evaluation, we will ask you for information about why you are choosing to leave. It is up to you whether to answer these questions.

**What if you have questions about this evaluation?**
You have the right to ask and receive answers to questions about this research. If you have questions, complaints, or concerns, contact the researchers listed below:

***Prof Geoffrey Setswe at 072 025 9875 or Jacqui Pienaar at 082 965 5098***

# OFFER TO ANSWER QUESTIONS

Before you sign this form, please ask any questions on any aspect of this evaluation that is unclear to you. You may take as much time as necessary to think it over.

# AUTHORISATION

I am making a decision about whether or not to participate in this evaluation. My signature indicates that I have read and understood the information provided above, have had all my questions answered, and I have decided to participate.

The date I sign the document to enrol in this evaluation, that is, today’s date, MUST fall between the dates indicated on the approval stamp affixed to each page. These dates indicate that this form is valid when I enrol in the evaluation but do not reflect how long I may participate in the evaluation.

**____________________________________ ___________**

Name of Research Participant (please print) Date

**_______________________________ ________________**

Signature of Participant Time in 24:00 format

**____________________ ___________________**

Names of Study Staff Signature of Study Staff

Obtaining Consent

**_______________________ ________________ ________________**

Name of Witness Signature of witness Time in 24:00 format

**STATEMENT OF CONSENT TO BE AUDIOTAPED**

I understand that audio recordings will be taken during the SMS input session and the evaluation interviews. *(For the statement below, please choose YES or NO by inserting your initials in the relevant box)*

- I agree to being audio recorded Yes

No

**______________________________ _________ ______**

Signature of Participant Date

**YOU WILL BE GIVEN A COPY OF THIS CONSENT FORM TO KEEP.**

If you have any questions concerning this evaluation or consent form beyond those answered by the investigator, including questions about the research, your rights as a research subject or research related injuries; or if you feel that you have been treated unfairly and would like to talk to someone other than a member of the research team. If you want any information regarding your rights as a research participant, or complaints regarding this evaluation, you may contact Prof. Clement Penny, Chairperson of the University of the Witwatersrand, Human Research Ethics Committee (HREC), which is an independent committee established to help protect the rights of research participants at (011) 717 2301.
