# Supplementary material for: When evidence is not enough: A qualitative exploration of healthcare workers’ perspectives on expansion of two-way texting (2wT) for post- circumcision follow-up in South Africa
Source: PLOS Digit Health. 2025 Jun 5;4(6):e0000867. doi: 10.1371/journal.pdig.0000867 (PMC12140269; doi:10.1371/journal.pdig.0000867)
Supplement: S3 File — Semi-structured interview guide for qualitative interviews. (DOCX) [file pdig.0000867.s003.docx]

**Key Informant Interview Guide to assess acceptability of 2WT among HCWs using CFIR.**

Adapted from: <https://www.ncbi.nlm.nih.gov/pmc/articles/PMC6248861/>

FIRST: Interviewer MUST verify that informed consent is signed by the interviewee

1. Verification of informed consent? CIRCLE ONE: YES NO

General introduction:

Hello, my name is ____________________. Thank you for agreeing to talk to me today as part of ongoing VMMC quality improvement efforts. This interview is about your experiences whilst using the two-way-texting follow-up method that your organisation has been implementing. We will explore and cover more of your thoughts, opinions, and suggestions about the texting study that you recently helped implement to inform improvements as we consider scaling up the follow-up approach. Thank you for agreeing to participate in this interview, this should take about 45 minutes of your time. I will be asking you some questions which you are free to answer in any way you wish. You may also choose not to answer any question. I encourage you to feel free to say anything concerning the topic of discussion. If a question is unclear to you, you can ask me to explain it. Your participation is voluntary and confidential. Whatever you tell me will be treated with utmost confidentiality. The information will only be used for the purposes of this program evaluation.

Please allow me to record our discussion so that I don’t miss anything. Your voice will not be heard by anyone other than our study team. Your name will not be recorded and will not appear on the transcription or any publication. The recording will be destroyed after we have prepared our transcripts.

Is it okay if I tape-record our discussion? YES NO [IF NO, STOP]

**SECTION A: For all interviewees**

1. To get started, I’d like to learn about your involvement with 2wT. Tell me about your role in the implementation. [**Characteristics of Individuals**]

**Probe:** for all roles (site (enrolment, messaging, reviews, M&E, hub, M&E, management, oversight, reporting, etc.)

**SECTION B: Only for site/hub/implementation**

1. Tell me about the initial 2wT training you received. [**Inner Setting]**
   1. What training materials were used to train you?

**Probe/show examples:** [be specific: did they use, like, dislike, not used]: posters, flip charts, pamphlets, user guide, (toolkit of client education), HCW cheat sheets, and short videos.

- 1. Which one was more useful to you?
  2. What were the most useful aspects of your training?

**Probe:** (PowerPoint slides training, educational materials, support phone calls)

- 1. Tell me about refresher training sessions or on-site mentoring/support you received AFTER the program started.
     - How was mentoring or support provided?

**Probe:** phone, WhatsApp, Zoom? In-person? Videos?

- 1. How can we improve the training?

**Probe:** Initial AND ongoing mentoring/support?

1. Now, let's talk about the implementation of the 2wT approach – you may not know all aspects, but let’s talk about your experiences. From your perspective, walk me briefly through the process from client education to enrolment to interaction over 14 days to reporting. At each stage, tell me what worked well and what did not. **[Intervention Characteristics]**
   1. Tell me about client sensitization on 2wT BEFORE a client is cut?

[How do you introduce 2wT?]

- - - Tell me about post-operative counselling? Overall, how can we improve client education?

- 1. How about with enrolment? What works well? What does not?
     - How long does the enrolment confirmation take?
     - Are there differences in SMS vs. WhatsApp?
  2. Regarding the Daily 2wT messaging, what worked well and what did not?
     - Following up on Potential AE reports
     - Communicating with clients
  3. Regarding the Task completion, what worked well and what did not? In your opinion, how easy or difficult is it to complete tasks?
     - For referrals to care
     - For tracing
  4. Regarding reporting and completion of the paper follow-up (review) forms, what works well and what does not? In your opinion, how easy or difficult is it to complete paper follow-up forms using information from the system?

How can we smoothen the challenges you experienced with paper and the 2wT interface? Then how can we smoothen that?

**SECTION C: For all interviewees**

1. Let’s talk about the effect of 2wT for VMMC at your clinic (your organization).

From your perspective as a HCW, **how does 2wT impact the quality of client follow-up?**

- - How does 2wT help find AEs?
  1. What is the impact of 2wT follow-up on demand creation? For HCWs, let’s talk about the impact of 2wT on your follow-up workload. How did 2wT impact your follow-up workload? [**Process**]

**Probe:** How could we further improve 2wT to reduce workload?

- 1. How could we improve 2wT for HCWs?

1. Tell me about how the hub nurse works with the site teams [Explain: Hub is the first line of nurse messaging and referral] **[Process of Implementation]**
   - Would you prefer the Hub nurse to be resident in your clinic or to be in a central location (one hub-to many spokes)? Why?
2. Let’s talk about scaling up. We’d like to learn about how 2wT could help more clients across more VMMC sites, in both routine and the busy season or campaign period.

Tell me about using 2wT during routine (10-20 clients per day) to peak campaigns – mass/peak season events.

- 1. How many 2wT clients do you think your clinic can manage at a time?
  2. Is this with a central hub or clinic-centred hub?
  3. What influences whether this number goes up or down?
  4. How could we improve 2wT for clients?
  5. How could we improve 2wT for nurses?
  6. How could we improve 2wT for data management?

1. Let’s talk about next steps.
   1. How can we make 2wT more user-friendly for workers?
   2. How can we make it more user-friendly?
   3. What new features or functions would you like to see in 2wT for MC?
   4. What are influential individuals like your immediate managers (for RtC staff) or your contractor (for GPs) saying about 2wT for VMMC? **[Process of Implementation]**
   5. Where else would you like to see 2wT used

**Probe**: Other healthcare contexts, locations, health areas?

1. Which opportunities do you think 2wT can bring to VMMC?
2. What concerns or threats do you think the implementation of 2wT can bring to VMMC?
3. Before we end, do you have anything else to add for the 2wT team to improve the implementation of VMMC/2wT services?
